# Human Papilloma Virus Vaccination: Focus on the Italian Situation

**DOI:** 10.3390/vaccines9121374

**Published:** 2021-11-23

**Authors:** Giovanni Gabutti, Erica d’Anchera, Francesco De Motoli, Marta Savio, Armando Stefanati

**Affiliations:** 1 Pro-Tempore National Coordinator of the Work Group “Immunizations and Vaccinal Policies” of the Italian Scientific Society of Hygiene, Preventive Medicine and Public Health (SItI), 16033 Lavagna, Italy; gbtgnn@unife.it; 2 Post-Graduate School of Hygiene and Preventive Medicine, University of Ferrara, 44121 Ferrara, Italy; dncrce@unife.it (E.d.); dmtfnc@unife.it (F.D.M.); svamrt@unife.it (M.S.); 3 Department of Medical Sciences, University of Ferrara, 44121 Ferrara, Italy

**Keywords:** HPV, HPV vaccination, papilloma virus, vaccines, adolescents, infectious diseases, cervical cancer, COVID-19

## Abstract

Human papilloma virus (HPV) is a viral agent whose transmission occurs mainly by sexual means. It causes different pathological conditions in both males and females, ranging from benign pathologies up to cancers. The introduction of vaccination has certainly had a major impact in terms of reducing the incidence of both HPV infections and diseases but in the European Union and the European Economic Area (EU/EEA) there are still about more than 13,000 deaths due to cervical cancer each year. To date in Europe and in Italy there are three vaccines available (bi-, tetra-, and nonvalent vaccines). The vaccination campaign started irregularly in Europe and Italy in 2007, with pre-adolescent girls as the primary target. Later, other cohorts were introduced such as 12-year-old boys, additional cohorts of >25-year-old women, women who already underwent cervical surgery and other subjects entitled to free vaccination. The COVID-19 pandemic has strongly impacted on public health services, particularly on vaccinations that, especially during the first pandemic phase, have been often delayed and/or canceled. The most affected vaccinations by the pandemic have been the non-mandatory ones, particularly those addressing the adolescent and adult population, such as immunization against papillomavirus. To date the achievement of the coverage target set by the Italian National Immunization Plan (NIP) has not yet been achieved. The aim of this work is to summarize the current situation in Italy and to discuss the strategies that have been implemented to increase overall vaccination coverage rates.

## 1. Introduction

Human papilloma virus (HPV) is an extremely widespread viral agent throughout the world; it includes more than 200 distinct genotypes and its human-to-human transmission occurs mainly by sexual means [1]. HPV causes different pathological conditions in both males and females, ranging from benign pathologies, such as warts, up to cancers of the cervix, vagina, vulva, anus, penis and oropharynx [2].

Worldwide, approximately 70% of cervical cancers and HPV-associated precancerous lesions are caused by the serotypes 16 and 18. Considering that this type of cancer is currently the fourth leading cause of death in the female world population, HPV is responsible for an important burden of disease [1].

In Europe, three different HPV vaccines are available differing each other in the number of serotypes they protect against [3]. In Italy, vaccination against HPV started in 2007 and over the years there has been an increase in vaccination coverage even if the target indicated in the 2017–2019 National Immunization Plan (NIP) has not yet been reached [4].

In Italy the COVID-19 pandemic has caused a sharp slowdown in immunization activities, particularly for vaccinations not included among the mandatory ones. The aim of this work is to summarize the current situation in Italy and to discuss the strategies that have been implemented to increase overall vaccination coverage rates as well as to allow catch-up interventions.

## 2. HPV Epidemiology in Europe and in Italy

HPV genotypes are numerous (more than 200) and some of them have a carcinogenic potential: HPV 16, 18, 31, 33, 35, 39, 45, 51, 52, 56, 58 and 59. HPV 16 is certainly the most widespread genotype in the world. The increased finding of neoplastic lesions in other areas of both genital (anus and penis as well as vulva and vagina) and extragenital tract (oropharynx), has raised attention on male subjects and on the benefit of a gender-neutral preventive approach [5].

Recent studies have found that in Europe the prevalence of HPV infection in women is 14%, taking into account the fact that the largest number of infections is found in adolescents and young adults, therefore in the more sexually active part of the population [5].

The latest report by the European Center for Disease Prevention and Control (ECDC) has shown that in the European Union and the European Economic Area (EU/EEA) there are currently about 34,000 new diagnoses of cervical cancer being 9.6/100,000 women the age-standardized incidence rate. Besides, >13,000 deaths are reported each year with a mortality rate of 2.8 per 100,000 women [5].

There are approximately 300,000–500,000 Cervical Intraepithelial Neoplasia grade 2 (CIN2) or more severe lesions identified by screening each year [5]. As regards the rest of the tumors of the anogenital tract, the number of cases per year amounts to 14,700, of which just over 60% are diagnosed in women (4200 in the anal and 5100 in the vulvar and vaginal tract) and the remainder in males (mainly in the anus and penis) [5].

Head and neck cancers have also a significant impact with an incidence of 13,800 cases per year, mostly (about 11,000) in males. A particularly at high-risk group of developing severe HPV disease is represented by HIV-infected individuals, particularly those highly immunosuppressed. Noteworthy, HPV 16 is the most prevalent genotype in Europe in HIV-positive women diagnosed with cervical cancer [5]. The introduction of vaccination against HPV has certainly had a major impact in terms of reducing the incidence of both HPV infections and diseases. In Norway, for example, after the introduction of quadrivalent vaccine, the prevalence of infection caused by the HPV genotypes contained in the vaccine decreased by 81% in 17 years old immunized women compared to the unvaccinated cohort [6]. In Switzerland, five years after the start of the vaccination program, it was found that the prevalence of HPV types included in the vaccine significantly decreased in <26 years old women [7].

Taking into account warts, it has been shown that in French women aged between 15 and 18 years, the incidence of genital warts significantly decreased in the period 2008–2012 after the introduction of the quadrivalent vaccine [8]; in Denmark, 5 years after the introduction of the same vaccine the incidence of genital warts decreased by 55% in women aged 12–35 years. Besides, in 12–29 years old men, the incidence of genital warts decreased by 37%, indicating the development of herd immunity [9]. In Spain, three doses of the 4-valent HPV vaccine guaranteed an efficacy of 76% against the incidence of genital warts in young (14–19 years old) women [10]. The introduction of the quadrivalent vaccine has also positively reduced the incidence of cervical cancer, as reported in Sweden and Denmark [11,12].

As for Italy, it is estimated that up to 80% of sexually active women contract a HPV infection during their lifetime, and that more than 50% become infected with a potentially oncogenic genotype [2]. In 2012 a systematic review showed a prevalence of 8% of oncogenic HPV genotypes in the general Italian population, with a peak in <25 years old women. A second peak in prevalence, albeit to a lesser extent, is observed in the post-menopausal age. In Italy too, HPV 16 is the prevalent genotype, present in 5% of the healthy population, followed by HPV 18 (about 1%) [2].

## 3. Vaccines Available in Europe and Italy, Primary Target Schedule and Differences between Italian Regions

Vaccines against HPV have been authorized by the European Medicines Agency (EMA) since 2006–2007: to date three vaccines are available: a bivalent vaccine containing HPV 16 and 18 genotypes, a tetravalent vaccine containing also HPV 6 and 11 genotypes and a nonavalent vaccine which contains genotypes 6, 11, 16, 18, 31, 33, 45, 52 and 58. This latter is used to protect against precancerous lesions and cancers in the cervix, vulva or vagina and anus, and genital warts caused by specific types of HPV [13]. These vaccines can be administered to both males and females [14]. All three vaccines include highly purified virus-like particles (VLPs) of the major HPV L1 protein and provide protection from HPV 16 and 18 genotypes associated with more than 90% of pre-cancerous lesions diagnosed in Europe [3]. HPV vaccines are formulated to induce humoral immune responses, are effective, well tolerate and safe (VLPs are unable to infect cells, reproduce or cause disease in vaccinated subjects) [13].

Accordingly to the age, the vaccination schedule provides for the administration of two doses at 0 and 6 months for subjects up to 13 or 14 years or the administration of three doses at the time 0, 1–2 and 6 months for subjects >14 years of age. The three-dose schedule is recommended even if the interval between the first two doses was less than five months or if the subject is suffering from immunosuppression [15,16].

All three licensed vaccines have proven their safety in clinical trials submitted at the time of their approval and are currently undergoing post-marketing monitoring by the EMA and other European national drug agencies confirming their safety [5].

Data emerging from the comparison between the pre- and post-vaccination period have highlighted the positive impact of European vaccination programs on the incidence of new HPV infections, on genital warts and high-grade cervical lesions in countries with high coverage rate. Furthermore, a cross-protection against genotypes not directly included in vaccines and herd immunity against non-vaccinated subjects has been shown [5]. Long-term data for quadrivalent vaccine administered in 16–23 years old women during the follow up at nine, ten and twelve years after administration have shown that no new cases of high-grade cervical lesions related to the serotypes included in the vaccine have been observed ten years after immunization and that no cases of CIN2 or vulvar or genital cancer have been observed in vaccinated women after twelve years following vaccination [17,18]. Besides, the immunogenicity results of a long-term study on quadrivalent vaccine have indicated sustained antibody responses against genotypes 6, 11, 16 and 18 up to fourteen years after immunization [19]. These conclusions are in line with the excellent results of an interim analysis (follow-up at 8 years) carried out on Scandinavian women vaccinated with the nonavalent vaccine; this analysis has confirmed the absence of high-grade lesions caused by the genotypes included in the vaccine in immunized women and the persistence over time of efficacy [19,20].

As for the male subjects, the long-term data are still being studied: the preliminary analysis regarding the follow-up at six and twelve years of 16–26 years old vaccinated subjects with quadrivalent vaccine indicate how the vaccine is extremely effective against high grade anal lesions and genital warts related to HPV genotypes included in the vaccine and how the ten-year seropositivity rates are comparable to the results obtained by evaluating the profile of women vaccinated with the same product [21]. Further confirmation of a decrease in the prevalence of HPV genotypes included in the quadrivalent vaccine has been observed comparing pre- and post-vaccine period in the vaccinated heterosexual male population in Australia and this decrease is most significant in infants younger than 21 years [22,23].

Starting from 2007, the states of the European Union implemented a specific vaccination program against HPV keeping adolescence as a primary target, firstly involving exclusively girls and more recently extending vaccination to boys and to some individuals with specific risk factors [5]. In Italy, since 2007 vaccination against HPV was proposed and recommended to 12-year-old girls as a primary target and additional age groups were proposed as a secondary target, such as 25-year-old women belonging to HPV screening services already subject to active call and a possible third cohort of women between the 12 and 25 years age cohorts [24].

Subsequently, the 2012–2014 NIP included among its objectives the achievement of a vaccination coverage for three doses HPV greater than or equal to 70% in 12-year-old girls starting from the 2001 cohort, ≥80% starting from the 2002 cohort, and finally ≥95% starting from the 2003 cohort [25].

In 2014 the Board of the Calendar for Life, subscribed by the Italian Society of Pediatrics, the Italian Federation of Pediatricians, the Italian Society of Hygiene, Preventive Medicine and Public Health and the Italian Federation of Family Doctors, published the proposal for an immunization calendar including the recommendation for vaccination against HPV also for twelve-year-old males [26].

Finally, the 2017–2019 NIP has included both sexes as primary targets for vaccination against HPV in adolescence, preferably before sexual debut. To date, the primary target is the immunization of the entire population (males and females) and the preferable age for the active and free offer of HPV vaccination in Italy is set at the 12th year of life [15].

As for all European countries, the start of the vaccination campaign in Italy was not uniform in the various regions of the peninsula: the first to start were the Basilicata and Valle d'Aosta regions in 2007 and then Tuscany, the Autonomous Province of Trento, Liguria, Veneto, Emilia-Romagna, Molise, Calabria, Lazio and Campania which started the vaccination campaign in the first part of 2008. Only in the last months of the same year Lombardy, the Autonomous Province of Bolzano, Friuli-Venezia Giulia, Umbria, Abruzzo, Puglia, Marche, Sardinia and Piedmont have aligned themselves with the vaccination offer for adolescent girls and today there is a free offer for the 12-year-old cohort of both sexes throughout the peninsula [27].

## 4. Multicohort Strategy: The Additional Cohorts of >25-Year-Old Women, Women Who Already Underwent Cervical Surgery and Other Subjects Entitled to Free Vaccination

The final goal of increasing the overall vaccination coverage rate against HPV of the entire Italian population can be achieved through the implementation of the so-called multi-cohort strategy, i.e., the vaccination of additional cohorts, in addition to the one already set as the primary target for boys and girls in their 12th year of life. The so-called secondary target includes the 25-year-old female cohort already involved in the active call for screening against cervical cancer; those belonging to this cohort could be invited to immunization on the occasion of the call for the HPV test. A possible third cohort of women eligible for a further active call is an intermediate one between the primary and secondary target (e.g., girls aged around 16 years of age to be vaccinated with a three-dose schedule) [15].

There are other subjects belonging to the so-called risk categories such as men who have sex with other men, sex workers, HIV-positive individuals, immunocompromised subjects or those who have to start therapy with immunomodulators and immunosuppressants, individuals with HPV-related genital lesions and women already treated for high-grade cervical neoplasms. Noteworthy, vaccinating women after cervical surgery can reduce the risk of recurrence and therefore limit any adverse event of repeated surgery and at the same time prevent new infections by HPV genotypes included in the vaccine [28,29,30].

There is also the rationale to vaccinate all >25-year-old women as adult women are particularly vulnerable to HPV infection, new HPV infections are acquired in adulthood due to changes in sexual life, immunosenescence can facilitate the reactivation of a latent infection or lead to a decreased ability to respond to natural infection, and there could be a cohort effect (high exposures throughout life occur in generations of older age) [15,28,31,32,33].

For these categories of people, the vaccination offer is varied and different in the Italian regions. In some Regions vaccination is free when directly requested by subjects belonging to at risk categories; in other Regions immunization is provided in co-payment. Nowadays, the free offer for women with previous HPV lesions and the free offer to 25-year-olds on the occasion of cervical cancer screening is currently available in 15 and in 14 Italian regions, respectively [34].

## 5. Current Vaccination Coverage Rates

According to the guide “Global strategies to accelerate the elimination of cervical cancer as a public health problem” published by the World Health Organization (WHO) at the end of 2020, 90% of 15-year-old adolescents in the world will have to be vaccinated by 2030 [35]. To achieve this goal, the offer of HPV vaccine must be introduced in all countries; in fact, as of June 2020, only 57% of the member countries of the United Nations had already introduced this immunization. The issue is not just to increase and optimize vaccination coverage, but to stimulate and support countries, especially low-income ones, to introduce and start administering HPV vaccine [36]. Up to June 2020, 85%, 77% and 56% of the states in America, Europe and Oceania and only 31% and 40% of states in Africa and in Asia had introduced vaccination against HPV, respectively. The disparity between high-, low- and middle-income countries is evident, as 88% of high-income states had introduced HPV vaccination for females in respect to 40% of low- and middle-income countries. HPV vaccination is offered to males in 44% of high-income countries and in 5% of low- and middle-income countries. The estimated global coverage with two vaccine doses at the end of 2019 was 15% for females and 4% for males and only 6% of United Nations members have reached the target threshold of 90% coverage [37].

According to a study published in 2019 collecting data on vaccination coverage from 30 EU/EEA countries and Switzerland in the period 2010–2017, the vaccination coverage rate was high (>71%) in ten countries (Norway, Finland, Sweden, Iceland, United Kingdom, Spain, Portugal, Belgium Flanders, Hungary and Malta), on average high (51–70%) in seven countries (Italy, Switzerland, Ireland, Denmark, Holland, Czech Republic, Luxembourg), low (31–50%) in four (Germany, Latvia, Slovenia and Belgium Wallonia) and very low (<30%) in four (Greece, France, Poland, Bulgaria). For some countries, data were not available [38].

**Figure 1:**
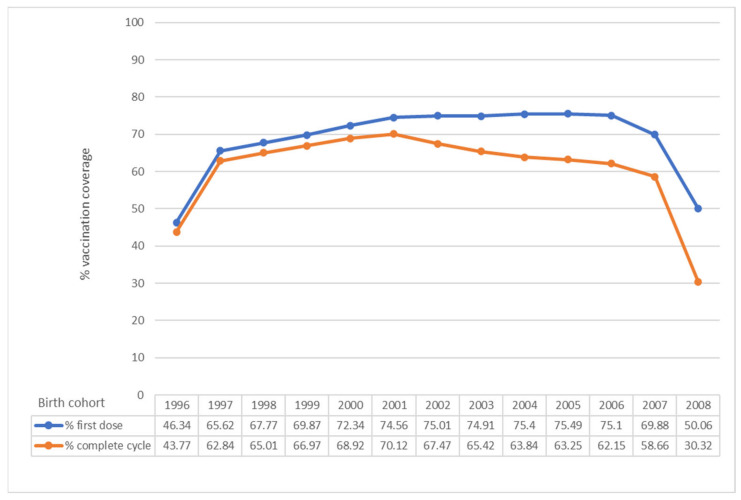
HPV vaccination coverage in Italy, observed year 2020. First dose and complete cycle per birth cohort (females). Data updated to 31 December 2020 (modified by [39]).

**Figure 2:**
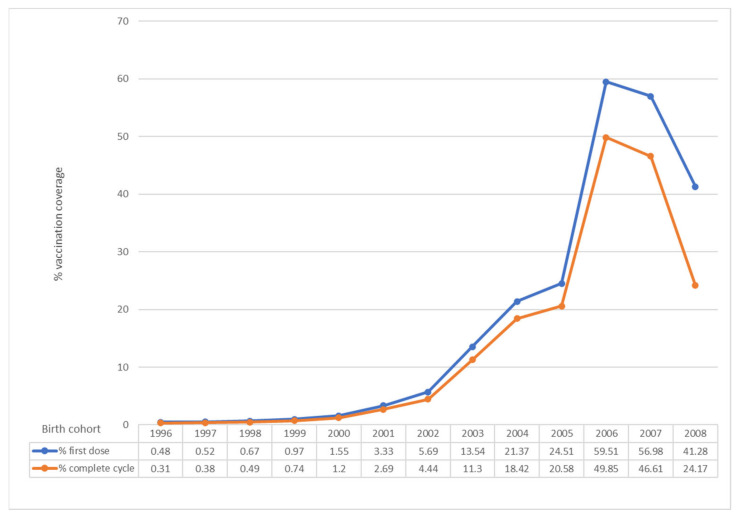
HPV vaccination coverage in Italy, observed year 2020. First dose and complete cycle per birth cohort (males). Data updated to 31 December 2020 (modified by [39]).

In Italy, an increase in vaccination coverage has been observed from 2007 to 2019, even if the target of 95%, set by the 2017–2019 NIP for both males and females has not been achieved in any region (Figure 1 and Figure 2) [4,39].

As regards females, the average HPV vaccination coverage is well below the optimal threshold provided by the 2017–2019 NIP (95% in the 12th year of life) (Figure 1). Only one cohort exceeds 70% for complete cycle (Figure 1). Also at the regional level, no Region has reached 95% in none of the evaluated cohorts [4,39].

**Figure 3:**
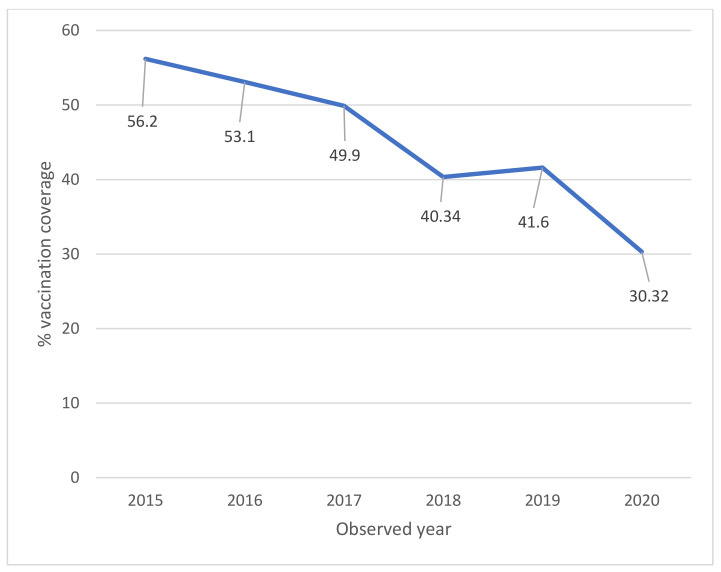
HPV vaccination coverage in Italy. Time trend for complete cycle per year (females). Data updated to 31 December 2020 (modified by [39]).

The full-cycle coverage for 11-year-old girls (2008 cohort in 2020) has shown a decrease compared to the full-cycle coverage for 11-year-olds of the previous years (30.32% in 2020 compared to 41.60% in 2019, 40.34% in 2018, 49.9% in 2017, 53.10% in 2016 and 56.20% in 2015) (Figure 3) [4,39].

For males, the average vaccination coverage rate is very far from the objectives set by the 2017–2019 NIP (95% in 2019) (Figure 2). Only two cohorts exceed 40% for complete cycle and none of them exceed 50% (Figure 2) [4,39]. Furthermore, a decrease has been observed in comparison to previous year as regards the complete cycle for the cohort of 11-year-olds (24.17% in 2020 compared to 32.25% in 2019). Instead, an improvement has been observed for the coverage of first dose for 11-year-olds (41.28% in 2020 compared to 39.08% in 2019) [4,39].

The data on vaccination coverage (full cycle) 2020, for both females and males, show a significant decrease compared to those referring to 2019 (Figure 1 and Figure 2). The decrease in coverage may be mainly due to organizational difficulties due to the management of the pandemic [4]. This data confirms what was found with the national survey promoted by the Ministry of Health to verify the impact of the COVID-19 emergency on vaccination activities, which found a decrease especially in the administration of the anti-HPV vaccine, particularly in some regions [4,40]. Full-cycle coverage for 15-year-old girls (used by WHO as a reference in its statistics) has been equal 63.84%, with a decrease compared to the previous year (70.35%) [4].

Targeted interventions would be necessary in specific geographical contexts bearing in mind that HPV vaccination, even not mandatory, is an Essential Level of Assistance and as such should be actively and free offered [4].

## 6. Impact of COVID on Coverage and Intervention Strategies

The COVID-19 pandemic, with the temporary forced block of many health activities including vaccinations and the fear of contagion in attending healthcare environments (especially during the first pandemic wave), has certainly had a significant impact on vaccination coverage rates. In Italy, vaccination against HPV is recommended but not mandatory: according to the data collected by the Ministry of Health through a questionnaire administered to Local Health Units, the age group 13 months–18 years has been the one with the greatest reduction in vaccinations during the first lockdown period in 2020, consequently, HPV turns out to be the one that has suffered the greatest decrease in coverage. Given the significant impact that the pandemic has had on public health, the Ministry of Health has outlined some specific strategies for the catch-up of vaccination activities such as the hypothesis of making telephone contacts with the families, the preparation of a list of cohorts of subjects not yet vaccinated, the preparation of information and awareness activities on the importance of vaccinations, the strengthening of communication campaigns and the expansion of vaccination services to reach the unvaccinated more easily. With regard to HPV vaccination, a particularly important task is carried out by the school and by the teachers, who during their daily contact with adolescents and their families can have on a central role in communicating and disseminating information about the importance of vaccinations [40].

Other useful tools for the catch-up and the maintenance of coverage rates are the involvement of general practitioners, pediatricians, gynecologists and midwives. A relevant role could be played by the active call of both sexes, the maintenance of the right to free vaccination at least up to 18 years of age and the right acquired based on the cohort of birth, the inclusion of subjects at risk in vaccine offer and the application of the previously listed multi-cohort strategies [15,31,41].

The role played by social media should also be considered central, especially in light of the infodemic period we are going through. Even before the pandemic, the important role of social media was highlighted as adolescents were the primary target of the HPV vaccination campaign [42].

A recent systematic review has identified the perceived low efficacy of the vaccine, possible side effects, lack of trust in health authorities and inadequate and partial information as the main causes of vaccine hesitancy regarding HPV vaccination in Europe [43].

Catch-up programs are essential to try to reach the coverage target set by the 2017–2019 NIP. According to a study published at the end of 2019, in Europe some of the countries that recorded the best vaccination coverage (>75%) in the period 2010–2017 had implemented mass catch-up strategies in a limited period of time, while in countries with low or very low vaccination coverage this strategy has not been implemented [38]. The target groups to which the catch-up program should be addressed may be different, depending on the epidemiological situation and the availability of resources in the country. Also according to this study, the free offer of the vaccine helps to implement and ensure good vaccination coverage [38], a strategy could therefore be to ensure that the vaccine is free for more cohorts, ideally for all.

The role of general practitioners and pediatricians is very important as they are the health professionals who first come into contact with adolescents and their families. Updated and continuous training on HPV vaccination are essential in order to keep these health professionals focused on this issue, so that they are constantly encouraged to talk and discuss with adolescents and their parents [44,45].

## 7. Conclusions

Vaccination coverage for HPV in Italy, according to updated data as of 31 December 2020, does not reach the 95% target set by the 2017–2019 NIP, both in male and female cohorts. Although over the years and until 2019 there has been an increase in coverage for female cohorts thanks to specific catch-up interventions and vaccination has also been recommended and made free for male cohorts, this has not been sufficient to guarantee the achievement of the target threshold. The use of additional cohorts and the implementation of multi-cohort strategies has certainly helped to increase the rate of citizens protected against HPV, but to date, not enough has been done to ensure effective coverage for the entire at-risk population. It is vital to continue pushing to ensure a gender-neutral approach to HPV vaccination. Providing adequate protection for all adolescents can lead to significant health gains for the general population, greater protection for the male population, especially for men who have sex with men who do not benefit from vaccination of the female population [46], and finally to a greater vaccination resilience [5].

Besides, a more homogeneous approach, both at European and national level, could help to reach the final target of 90% for 15-year-old girls set by the WHO for 2030. In European countries, in fact, a great variation of the HPV vaccination coverage is registered [37], which is not the case for other vaccinations (e.g., diphtheria, polio, tetanus and measles) [38].

Last data have shown the terrible impact that COVID-19 has had on HPV vaccinal coverage rate. In this historical moment in which vaccines are a topic of daily discussion, it is of fundamental importance to guarantee and disseminate information that is as clear, simple and reliable as possible and that is aimed at both adolescents and parents. Furthermore, given that when we talk about HPV vaccination it is inevitable to also talk about sexual health, there is the opportunity to deliver scientifically correct information on sexually transmitted diseases and sexual education in general.

It is therefore essential, especially after the impact of COVID-19 pandemic, to try to implement even more expanded catch-up interventions and to continue with vaccination programs involving more and more professionals close to adolescents and to their families such as general practitioners, pediatricians, gynecologists, midwives, and teachers.

## Data Availability

No new data were created or analyzed in this study. Data sharing is not applicable to this article.
